# Easy Nitrite Analysis of Processed Meat with Colorimetric Polymer Sensors and a Smartphone App

**DOI:** 10.1021/acsami.2c09467

**Published:** 2022-08-03

**Authors:** Marta Guembe-García, Lara González-Ceballos, Ana Arnaiz, Miguel A. Fernández-Muiño, M. Teresa Sancho, Sandra M. Osés, Saturnino Ibeas, Jordi Rovira, Beatriz Melero, Cesar Represa, José M. García, Saúl Vallejos

**Affiliations:** † Departamento de Química, Facultad de Ciencias, Universidad de Burgos, Plaza de Misael Bañuelos s/n, 09001 Burgos, Spain; ‡ Departamento de Biotecnología y Ciencia de los Alimentos, Universidad de Burgos, Plaza de Misael Bañuelos s/n, 09001 Burgos, Spain; § Departamento de Ingeniería Electromecánica, Escuela Politécnica Superior, Universidad de Burgos, Avenida Cantabria s/n, 09006 Burgos, Spain; ∥ Universidad Politécnica de Madrid, 28040 Madrid, Spain

**Keywords:** polymer chemosensory films, sensors, nitrite in meat, colorimetry, RGB, HSV, color space

## Abstract

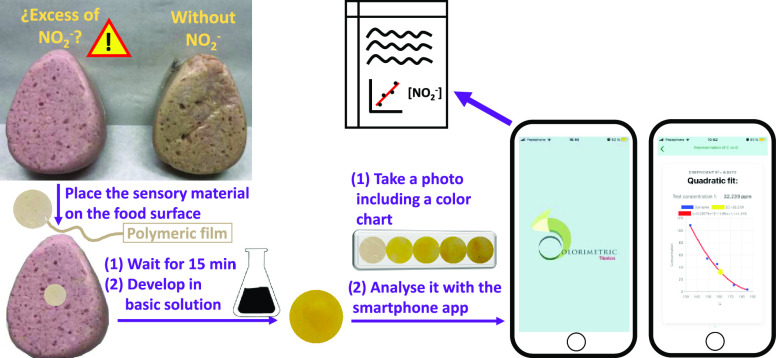

We have developed an in situ methodology for determining nitrite concentration in processed meats that can also be used by unskilled personnel. It is based on a colorimetric film-shaped sensory polymer that changes its color upon contacting the meat and a mobile app that automatically calculates the manufacturing and residual nitrite concentration by only taking digital photographs of sensory films and analyzing digital color parameters. The film-shaped polymer sensor detects nitrite anions by an azo-coupling reaction, since they activate this reaction between two of the four monomers that the copolymer is based on. The sensory polymer is complemented with an app, which analyzes the color in two different digital color spaces (RGB and HSV) and performs a set of 32 data fittings representing the concentration of nitrite versus eight different variables, finally providing the nitrite concentration of the test samples using the best fitting curve. The calculated concentration of nitrite correlates with a validated method (ISO 2918: 1975) usually used to determine nitrite, and no statistically significant difference between these methods and our proposed one has been found in our study (26 meat samples, 8 prepared, and 18 commercial). Our method represents a great advance in terms of analysis time, simplicity, and orientation to use by average citizens.

## 1 Introduction

The interest of society in having a balanced and healthy diet has increased remarkably in the last two decades.^1−4^ As a direct consequence, there is a need to detect and control different chemical compounds added to processed food, such as processed meat.^5^

**Figure 1:**
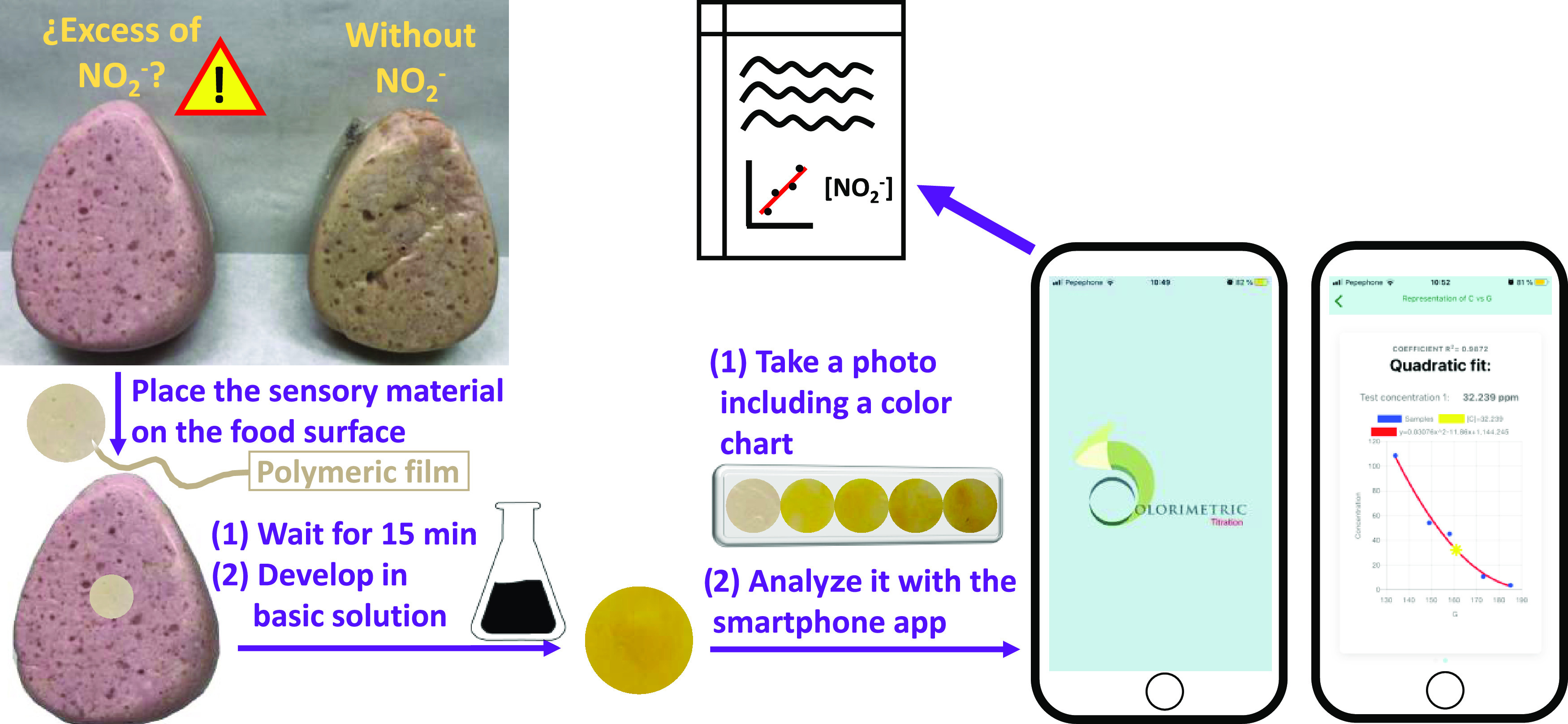
Proposed methodology for the one-pot determination of the nitrite concentration in food samples. The new method is based on a film-shaped polymeric film and is powered by the smartphone application Colorimetric Titration.

One of the most worrying additives is nitrite, commonly used as a preservative in food products such as processed meat,^6−9^ which provides a characteristic pinkish color and fresh meat flavor to products, e.g., york ham or mortadella (see Figure 1). However, nitrite anions are directly related to gastrointestinal tumors, stomach cancer, and the so-called blue baby syndrome.^6,8,10^ The World Health Organization (WHO) has set a fatal nitrite dose of 3 mg L^–1^ (65.2 μM) in drinking water. In the human body, concentrations from 33 to 250 mg of nitrite per kg of body weight are lethal and from 0.4 to 200 mg kg^–1^ of body weight are enough to cause methaemoglobinaemia.^11^

With this background, there is high interest in controlling the nitrite amount in foodstuffs, especially by the final consumer,^12−14^ since due to nitrite’s reactivity with hemoglobin and other products, the residual level of nitrite varies over time.^15,16^ In general, actual methods for determining the nitrite concentration are not oriented to the use by average citizens, are tedious, require reagent manipulation by specialized personnel, and use laboratory equipment (such as fluorimeters or spectrophotometers).^6,17−19^ Most relevant advances in this field described in the bibliography depict devices based on light-emitting diodes and photodiodes.^20,21^ Despite being increasingly simple systems, they are still dependent on a battery, an electrical circuit, etc., so their application in real life is not obvious, especially in food control, food safety, or food packaging. Electrochemical systems are also a family of powerful analytical techniques but present also the same disadvantages.^22^

This work describes a sensory polymer sensitive to nitrite anions,^23^ as a colorimetric sensory film (POLYSEN) for the visual detection of nitrite by simply putting it in contact with the food surface for 15 min and then immersing it in a basic solution. Going further to visual detection, color changes registered with a smartphone digital camera are an increasingly proven tool to quantify species of interest,^24−26^ including nitrite.^27,28^ However, we have realized that the end user must analyze and often interpret the results. Again, this is a barrier for most citizens, so we have boosted the usability of our nitrite quantification system with the development of an application for Android and iOS “Colorimetric Titration” (freely available from App Store and Google Play),^29,30^ which automatically autocalibrates the measurement, analyzes the data, and finally outputs a nitrite concentration result to the user.

In short, the novelty of our proposal for analyzing nitrite in meat lies in the combination of the following characteristics: (1) a costless novel sensory material based on a high amount of a hydrophilic monomer (*N*-vinyl-2-pyrrolidone, mol 45%), which ensures the absorbent properties of the material, and a hydrophobic monomer (methylmethacrylate, mol 45%), which ensures that the material can withstand handling by nonspecialized personnel; (2) an easy experimental procedure by only touching and revealing; (3) self-calibrated measurement; and (4) a novel smartphone application that allows the in situ measurement that can be easily used by the average persons (Figure 1).

## 2 Material and Methods

### 2.1 Meat Samples

This methodology was initially validated with cooked pork shoulder samples prepared on a pilot plant. Once the procedure was optimized, commercial samples of different products containing varying percentages of meat pork were also tested.

#### 2.1.1 Prepared Meat Samples

A total of eight representative samples of cooked pork shoulder (five samples to calibrate the system, and three test samples, termed T1, T2, and T3) were prepared. The nitrite concentrations added in the manufacturing were 1.0, 37.5, 112.5, 150.0, and 300.0 mg kg^–1^. Additionally, replicates of 300.0 (T1), 37.5 (T2), and 1.0 mg kg^–1^ (T3) were prepared as test samples.

For the elaboration of meat products in the pilot plant, pork shoulder trimmings were used. The fat was removed from the trimmings to obtain lean meat and was minced with a 15 mm plate in a meat grinder. A total of 8 kg of meat mass was made by vacuum mixing lean meat as the starting mix with the composition depicted in Table S1 (Section S2 in the SI). Following, this mass was split into eight batches of 1 kg, and different amounts of sodium nitrite, sodium chloride, carrageenan, starch, and water were added according to Table S2 (Section S2 in the SI) and all mixed together. Each batch’s meat mass was vacuum-sealed in a heat-shrinkable plastic bag, clipped, and after short-term dipping in hot water, was put in stainless steel molds before cooking in a meat oven at 80 °C till the product reached 72 °C in the center of the meat piece. Once cooked, products were kept for 24 h in refrigeration before unmolding. This process, especially the mixing, was optimized to obtain very homogeneous samples regarding composition and surface texture.

#### 2.1.2 Commercial Meat Samples

Once the method was optimized with the prepared samples, 18 different meat products were purchased to carry out a proof of concept. The products were purchased in two different supermarkets in northern Spain (additional information is in Section S7 in the SI).

### 2.2 Materials

All materials and solvents were commercially available and used as received unless otherwise indicated. The following materials and solvents were used: methylmethacrylate (MMA) (Merk, 99%), *N*-vinylpyrrolidone (VP) (Acros Organic, 99%), 4-aminostyrene (SNH2) (Aldrich, 99%), 3-aminophenol (Aldrich, 99%), methacrylic anhydride (Aldrich, 99%), hexane (Aldrich, 99%), hydrochloric acid (VWR-Prolabo, 37%), sodium hydroxide (VWR-Prolabo, 99%), sodium nitrite (Applichem Panreac, 99%), ethanol (Aldrich, 96%), zinc acetate dihydrate (Aldrich, 99.5%), acetic acid glacial (Aldrich, 100%), potassium hexacyanoferrate (II) trihydrate (Aldrich, >98.5%), sulfanilic acid (Aldrich, 99%), and 1-naphthylamine (Merck, 99%). Azo-bis-isobutyronitrile (AIBN, Aldrich, 98%) was recrystallized twice from methanol.

### 2.3 Instrumentation

Digital pictures were taken of the sensors (8 mm diameter discs) with an iPhone 8 smartphone (Apple Inc., Cupertino, CA) and processed with the developed app. For the proof of concept (Section 3.4), an additional smartphone was used (Samsung Galaxy Note 20 Ultra) to demonstrate the methodology′s versatility and the non-dependence on a specific smartphone. The app can be freely downloaded from the App Store and Google Play.^29,30^ UV–vis spectra were recorded using a Hitachi U-3900 UV–vis spectrophotometer (Hitachi High Technologies Corporation, Tokyo, Japan). Infrared spectra (FTIR) were recorded with an infrared spectrometer (FT/IR-4200, Jasco, Tokyo, Japan) with an ATR-PRO410-S single reflection accessory. High-resolution electron-impact mass spectrometry (EI-HRMS) was carried out on a spectrometer (Micromass AutoSpec Waters mass, Micromass Holdings Ltd., Cary, North Carolina) using an ionization energy of 70 eV and a mass resolving power >10 000. ^1^H and ^13^C{^1^H} NMR NMR spectra (Avance III HD spectrometer, Bruker Corporation, Billerica, Massachusetts) were recorded at 300 MHz for 1H, and 75 MHz for 13C, using deuterated solvents like dimethyl sulfoxide (DMSO-*d*_6_) or deuterated chloroform (CDCl_3_) at 25 °C.

### 2.4 Design and Synthesis of the Film-Shaped Polymeric Sensor (POLYSEN)

The polymeric material was designed as a film with gel behavior.^31^ In previous works,^32^ we have shown that this type of sensory material can react effectively with targets in the solid state. This property makes this format ideal for the proposed application, in which the film is simply left on the food’s surface, without the need to carry out the detection using a solvent.

The sensory film was prepared by bulk radical polymerization of three commercial monomers (VP, MMA, and SNH2) and one synthetic monomer (*N*-(3-hydroxyphenyl)methacrylamide, HPMA) in a molar feed ratio of 45:45:5:5 (VP/MMA**/**SNH2**/**HPMA) using 1% weight of AIBN as a radical thermal initiator. The synthesis and characterization of HPMA are depicted in Section S3 in the SI. In an oxygen-free atmosphere, the polymerization was carried out overnight at 60 °C, in a mold comprising two silanized glasses (100 μm thick). The sensory film was removed from the mold, washed three times with methanol, dipped in HCl (4%) for 30 min, and thoroughly washed with water to eliminate the excess acid. This acid treatment allows the formation of the nitrosyl cation in the presence of nitrite. Sensory discs, ϕ 8 mm, were punched from POLYSEN. The complete characterization of the material is depicted in Section S4 in the SI.

### 2.5 Methods

#### 2.5.1 ISO 2918:1975 Reference Method for the Detection of Nitrite Anions

In this standardized method, the food sample is extracted by stirring in hot ethanol for at least 1 h and treated with Carrez reagents. The solution is shaken, let stand for 10 min, and finally centrifuged for 5 min at 2000 r.p.m. to separate the fat and evaporate the liquid to a final volume of 200 mL.

After decolorization with activated charcoal and the addition of the colorimetric reagent (sulfanilic acid and α-naphthylamine), the solution is allowed to stand in the dark for 15 min at room temperature. Finally, the absorbance is measured at a wavelength of 520 nm and the analysis time for one meat sample was around 150 min.

All samples, both prepared and commercial ones, were tested with this method. The eight prepared meat samples were measured 9 days after their preparation, and the commercial samples were bought and measured on December 10th and 14th, 2021.

The calibration curve was carried out in the same way, using prepared aqueous sodium nitrite solutions at different concentrations. More information about the standardized procedure can be found on standard ISO 2918:1975 and in Section S1 in the SI.

#### 2.5.2 POLYSEN + APP Method for the Detection of Nitrite Anions

The proposed method is based on three main steps: (1) the preparation of the color chart; (2) the taking of the digital photograph, including the test sample and the color chart; and (3) the measurement using the app.

First, one POLYSEN disc was placed on the surface of each prepared meat sample (five prepared meat samples as calibration samples) with different nitrite concentrations for 15 min at room temperature. After that, the POLYSEN discs were dipped in an aqueous 0.1 mol L^–1^ NaOH solution for 1 min at room temperature. POLYSEN discs were plasticized as a color chart, which is, in this case, specifically designed for cooked pork shoulder samples and stable over time (at least for 1 year). The calibration meat samples must always be measured in parallel with the reference method, but this step is mandatory only once and is expected to be carried out by the manufacturer of POLYSEN in a real situation, not by the end user.

**Figure 2:**
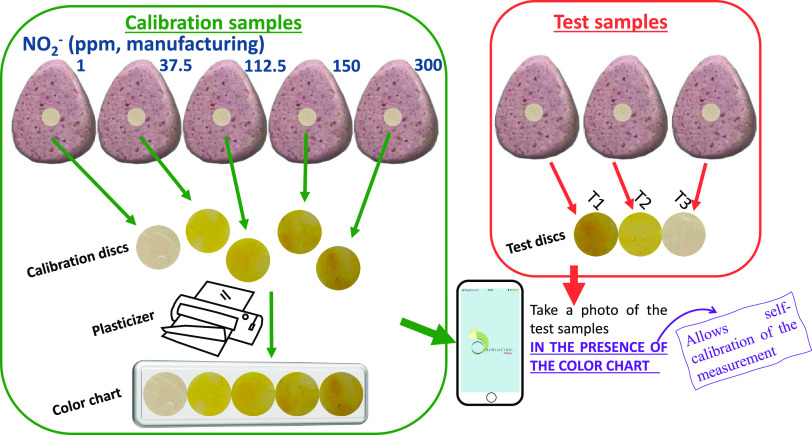
Image of different POLYSEN discs (five titration POLYSENs and three test POLYSENs) after being in food contact for 15 min and then developed in an aqueous 0.1 mol L^–1^ NaOH solution for 1 min. Then, the titration samples are plasticized as a color chart and finally photographed together with the test samples.

Second, the test samples were measured in the same way, i.e., by placing them on the meat surface for 15 min and revealing them in NaOH. The color chart and the test samples were photographed together with an iPhone 8 (see Figure 2). This step is expected to be carried out by the end user in a real situation.

Third, the taken photograph was analyzed with the app. The proposed method is based on taking a joint photograph of the calibration color chart (prepared by the manufacturer) and the test POLYSEN discs (tested by the end user). In this way, the system self-calibrates in each measurement, since the conditions for image acquisition are exactly the same for both the calibration color chart and the test POLYSEN discs. This fact makes the method not dependent on the smartphone model, the lighting conditions, or the distance to the object.

**Figure 3:**
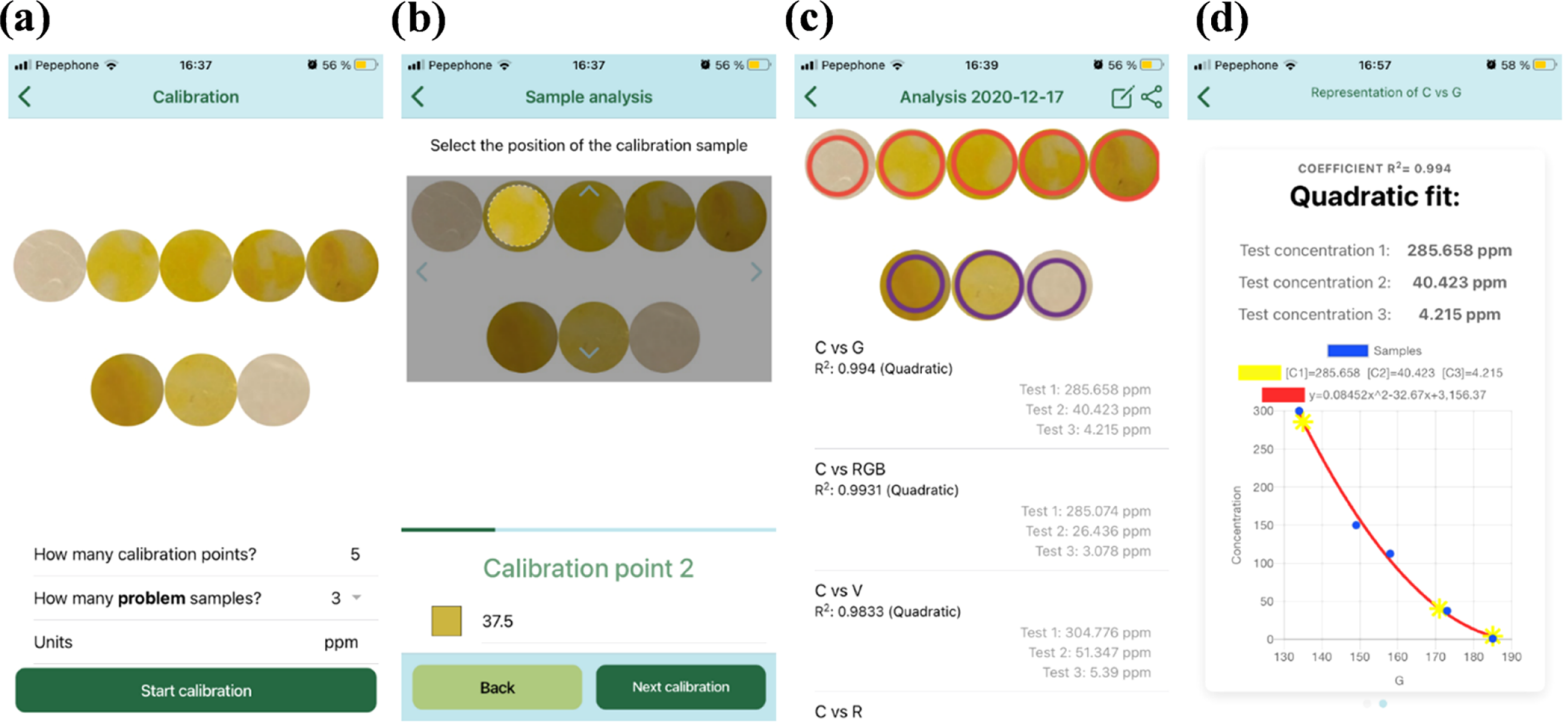
(a) Screen shown in the app after the photograph is uploaded, asking for the number of titration and test samples. (b) In the screen shown in the app, the user has to adapt the circular selector to each POLYSEN disc and enter the known concentration value. In the case of test samples, the user has only to adapt the circular selector. (c) Screen shown in the app after performing 16 linear and 16 quadratic fits of concentration (and the logarithm of concentration) versus different digital color parameters. The fit with the highest *R*^2^ parameter is chosen as the best option and is shown in the first position. (d) Final screen shown by the app after clicking on each fit. The app shows the graph with the titration (blue) and test (yellow) points, as well as the calculated nitrite concentrations for the test samples. All data and curve fittings can be shared in spreadsheet format (CSV).

Once the photograph is taken, the application first asks for the number of discs used for the calibration color chart and the number of test POLYSEN discs (Figure 3a). After that, the user has to adapt the circular selector to the size and position of each disc of the calibration color chart and then enter the concentration values that were initially added by the manufacturer (Figure 3b). Finally, the user must adapt the circular selector to the test POLYSEN discs. At this point, the application reads the different digital color parameters (red R, green G, blue B, hue H, saturation S, and value V). Additionally, using the read RGB parameters, the principal components of RGB^33^ and ΔRGB^34^ are also calculated. In total, eight different variables are selected. The application performs the graphical representation of the entered concentration against the eight variables (Figure 3c), also builds the graphs with the logarithm of the concentration, and performs linear and quadratic fits. In total, the application makes 32 fits, and the fitted curve with the highest *R*^2^ coefficient is considered the best option; thus, it appears in the first position. This part of the work was performed with an iPhone 8, a calibration color chart of five points, and three test POLYSEN discs. Taking no care of illumination (standard lighting conditions), the nitrite concentration versus green (G) parameter of digital color reaches the highest *R*^2^ coefficient. Thus, this is the fitted curve equation the app uses to calculate the concentrations of the test samples, as shown in Figure 3d. For more information about the use of the application, see Section S5 in the SI.

After these considerations, it is worth mentioning that the photographs containing the same calibration color chart, with the same test POLYSEN disc, but made in other light conditions (such as in a dark room, using the camera flash), or taken with another smartphone model, will indeed show different colors and therefore different parameters. However, as far as the end user is concerned, it does not imply any problem because the app will simply calculate the concentration of the test sample with another equation that fits better in those lighting conditions, smartphone model, camera, etc.

### 2.6 Migration Tests

The migration study was carried out as described in Commission Regulation (EU) No. 10/2011 (and amendments) relating to plastic materials and articles oriented to food contact applications.

The standard test methods were carried out at 100 °C with acetic acid, ethanol, and olive oil (EN 1186-3:202, 2002; UNE-EN 1186-1:2002, 2002).^35,36^

### 2.7 Limits of Detection (LOD) and Quantification (LOQ)

The limit of detection (LOD) and the limit of quantification (LOQ) of our sensory system was calculated by the following equations: LOD = 3.3 × SD/s and LOQ = 10 × SD/s, where “SD” is the standard deviation of the blank sample and “s” is the slope of a calibration curve in the region of low analyte content (below 21 ppm; more information is in Section S6 in the SI).

## 3 Results and Discussion

### 3.1 Nitrite Detection Principle

**Figure 4:**
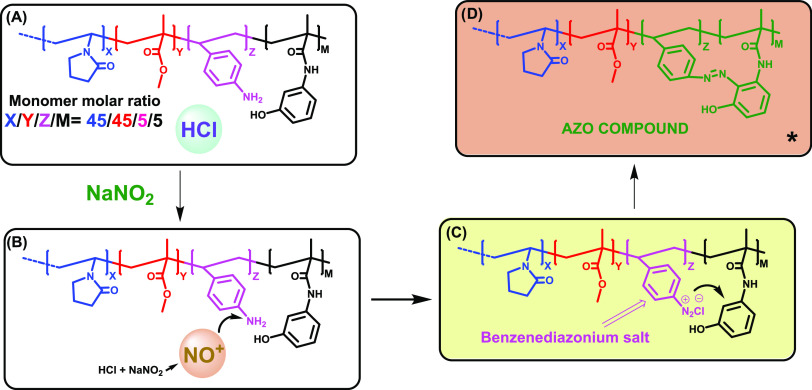
Film-shaped polymeric sensor’s (POLYSEN) chemical structures in the different stages of the detection process: (A) starting material, (B) nitrosyl cation formation inside the film, (C) benzenediazonium salt formation, and (D) azo compound formation. *The formation of the azo compound is also possible in the *p*-OH position, but only the *o*-OH substitution is shown for clarity.

Nitrite detection is based on the widely known and studied azo coupling, both in solution and in the solid phase.^31,37^ This irreversible reaction is activated by nitrite anions and is based on three stages, as shown in Figure 4. First, nitrite anions react with hydrochloric acid contained in the material and form the highly reactive nitrosyl cation. In the second stage, nitrosyl cations react with aniline units provided by the SNH2 monomer, resulting in the formation of a benzenediazonium salt derivative. Immediately, this benzenediazonium salt derivative reacts with phenol units provided by the HPMA monomer, allowing the formation of the yellow-red azo compound. This straightforward concept, combined with the digital color analysis performed with the app, results in a simple method for the detection of nitrite with a LOD of 0.85 ppm and a LOQ of 2.57 ppm.

Since the hydroxyl group of the HPMA monomer activates both ortho and para positions, the azo-coupling reaction could occur in three different positions, the *p-*OH position being the most favored one. In fact, the synthesis and characterization of very similar compounds (valid as models) have already been published, both in *p*-OH and *o*-OH positions.^38,39^

Although only 10 mol % of the monomeric units are reactive in the presence of nitrite, the rest of the material has a very high relevance, especially concerning stability over time, handling, and hydrophilicity. As shown in previous works, this type of material allows stabilizing compounds as reactive as benzenediazonium salts, allowing these sensors to be used months after their manufacture without losing activity.^31^ In addition, the molecular recognition of the sensory motifs toward the target is improved in this type of solid material compared to the same recognition in liquid solutions, due to the protective effect exerted by the polymeric environment.^40^ Finally, the combination of hydrophilic and hydrophobic monomers allows working on 100% aqueous media, keeping good manageability/handling even in the swelled state.

### 3.2 Migration Study

Nitrite detection implies that the material contacts the surface of the foodstuff, as plastics in everyday packaged meat products. Thus, the material was tested under the same regulations for ensuring the nonmigration of any substance (toxic or not toxic) to the foodstuff. The results show that POLYSEN complies with the restriction for the overall migration limit (<10 mg dm^–2^) as defined in the mentioned European regulation. Specifically, migration results obtained for POLYSEN in 3% acetic acid, 10% ethanol, and olive oil were 0.7, 5.9, and 4.8 mg dm^–2^, respectively.

### 3.3 Validation of the Sensory Polymer with the Prepared Meat Samples

The results of nitrite concentration calculated by both the reference and POLYSEN + APP methods are shown in Table 1.

**Table 1:** Obtained Data for Nitrite Concentration of Test Samples Calculated with the Reference Method (ISO 2918:1975) and the POLYSEN + APP Method^a^

	residual [NO_2_^–^] (mg kg^–1^)	
test samples	ISO 2918:1975 (reference method)	POLYSEN + APP method
T1	108.62 ± 0.12	108.01 ± 4.32
T2	10.59 ± 0.16	13.12 ± 1.91
T3	3.57 ± 0.08	2.90 ± 0.27

a Nitrite concentration data from the reference and POLYSEN + APP methods are means of ± standard deviation of 2 and 3 replicates, respectively.

For the proposed method, the application was run with the photograph containing the calibration color chart and test POLYSEN discs shown in Figure 2. While the manufacturing nitrite concentrations of the prepared meat samples were 1.0, 37.5, 112.5, 150.0, and 300.0 ppm, the obtained residual concentrations were 3.57 ± 0.08, 10.59 ± 0.16, 45.23 ± 0.31, 54.16 ± 0.01, and 108.62 ± 0.12 ppm, respectively. Thus, we used these residual values, since we think they are more realistic. All nitrite values are reduced after manufacturing due to their different compounds, such as hemoglobin, except for the sample made with 1 ppm nitrite, which increases to 3.57 ppm. Our interpretation is that it is the basal nitrite concentration, since the value obtained from a blank sample provided a very similar result.

The best fit resulting from the analysis was the [NO_2_^–^] versus G parameter (green), obtaining an *R*^2^ coefficient of 0.992. Fits with other parameters could also be good, and it is the end user who chooses the best, or the most appropriate, or simply the one with the higher *R*^2^ coefficient value. For that, the app can export all results as a “CSV” file that can be found in ESI-APP-DATA. Once selected the best fit, the application calculates the nitrite concentration of the test samples (Table 1).

As shown in Table 1, the proposed POLYSEN + APP method gives very similar values to the reference method, i.e., the proposed method could replace the reference method for quantifying nitrite in food samples. Statistical analysis also supports these results (see Section S8 in the SI), in which no statistically significant differences have been found between the reference method and the POLYSEN + APP method.

### 3.4 Proof of Concept: Working with Commercial Samples

We describe a new method for detecting nitrite anions aimed to be used as home analysis by nonskilled citizens. Thus, we believe a real example would be clarifying for the reader. Therefore, we pose a fictitious situation (based on completely real measurements) in which a manufacturer of polymeric materials starts manufacturing the “POLYSEN KIT”.

#### 3.4.1 Case Study A

**Figure 5:**
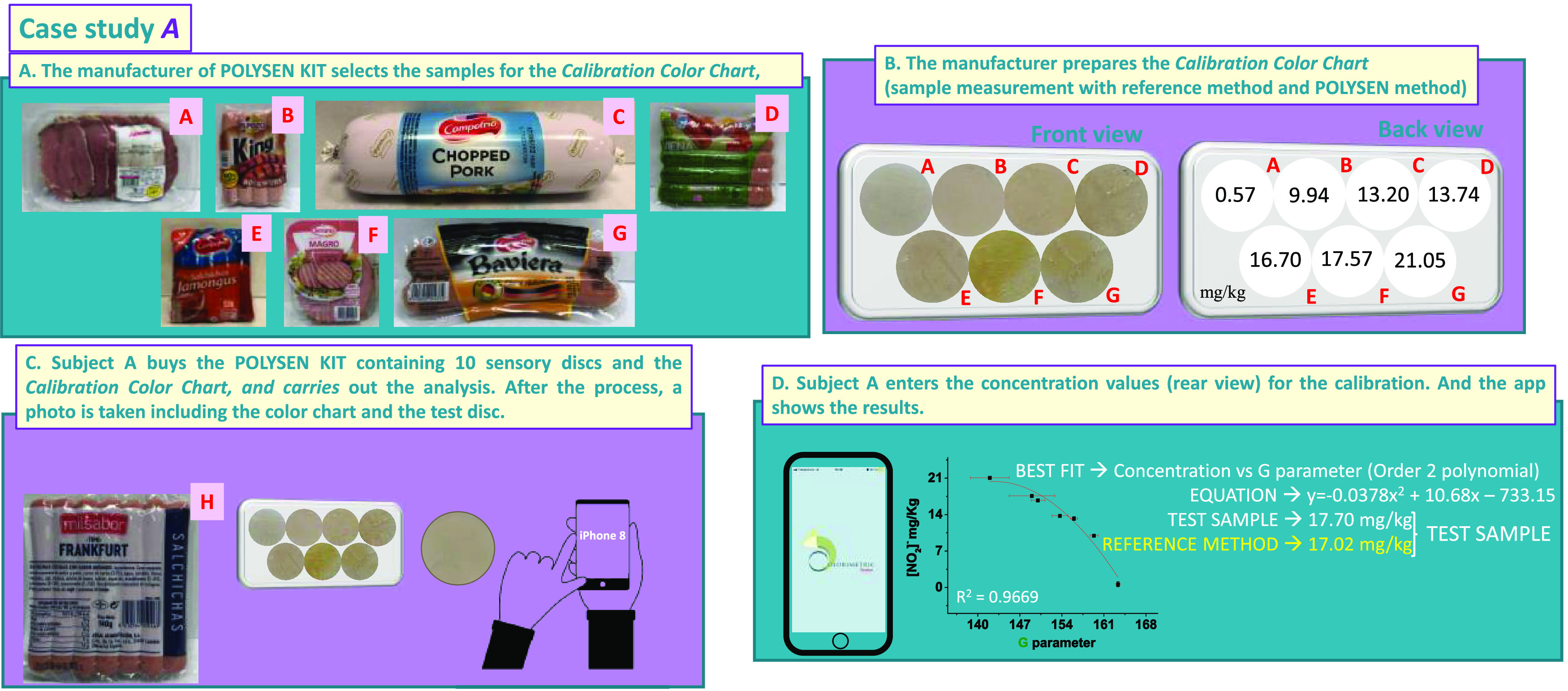
Case study A of the proof of concept. (a) Choice of samples for the preparation of the calibration color chart; (b) calibration color chart, front and back view; (c) example of the measurement of a problem sample with an iPhone 8 and a POLYSEN disk; and (d) results of the test sample obtained by the app Colorimetric Titration (the concentration value obtained by the reference method is also displayed).

This case study was carried out with the iPhone 8 of subject A, and a color chart containing seven points, which was prepared according to Section 2.5.2, but in this case with seven commercial samples of different products (Figure 5a,b).

Subject A took a small portion of the product he wanted to analyze and left a POLYSEN disk on its surface for 15 min. After developing the disc in basic water, he took a joint photograph of the color chart and the test disc and analyzed it with the Colorimetric Titration app (Figure 5c).

The best fit result was the one that appeared first (concentration vs G), and the nitrite concentration result was 18.3 mg of nitrite per kg (Figure 5d).

#### 3.4.2 Case Study B

**Figure 6:**
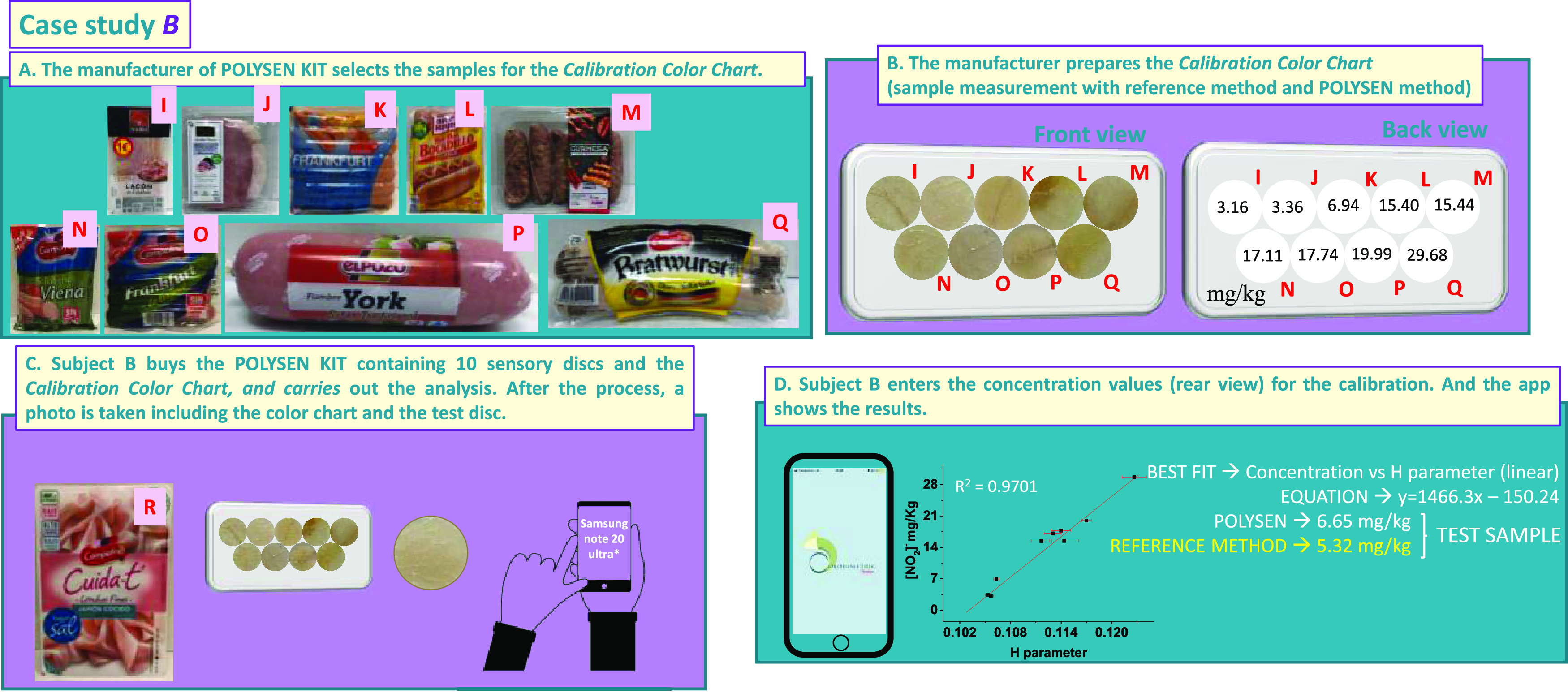
Case study B of the proof of concept. (a) Choice of samples for the preparation of the calibration color chart; (b) calibration color chart, front and back view; (c) example of the measurement of a problem sample with a Samsung Note 20 Ultra 5G and a POLYSEN disk; and (d) results of the test sample obtained by the app Colorimetric Titration (the concentration value obtained by the reference method is also displayed).

This case study was done with a different smartphone, Samsung Note 20 Ultra 5G, and completely different commercial products. Specifically, the color chart was made with nine commercial products (Figure 6a,b).

Subject B followed the same procedure as subject A, but the joint photograph of the color chart and test disc was taken under different light conditions, with another smartphone model, and at a different distance to the object.

After the analysis with the app (Figure 6c), subject B reached the best fit when representing [NO_2_^–^] versus the “H” parameter (from HSV color space: hue, saturation, value). The nitrite concentration result was 6.3 mg of nitrite per kg (Figure 6d).

To draw solid conclusions about the results of this proof of concept, we measured the nitrite concentration of the two test samples of the two case studies with the reference method. As shown in Figures 5d and 6d, the results are very similar to those obtained with the POLYSEN KIT. Specifically, the nitrite concentrations obtained with the reference method were 17.0 and 5.3 mg kg^–1^ for case studies A and B, respectively.

Additionally, a statistical analysis of the results was performed (Section S8 in the SI), and no significant differences were found between both methods.

## 4 Conclusions

A new sensory polymeric film-shaped material has been developed to detect nitrite in meat products through a color change. The procedure is as simple as leaving the material on the meat surface, waiting for 15 min, and developing it in aqueous NaOH (1 M). This research work is designed so that unskilled citizens could use this sensor, since the developed application for smartphones quickly analyzes the color change and gives a nitrite concentration value. One of the weak points when using photograph-based analysis has been overcome: the conditions in which the photographs are taken. First, the calibration POLYSEN discs must be plasticized once the calibration samples have been measured to obtain a calibration color chart stable over time. Second, the photograph of the test samples must always be taken in the presence of the color chart. In this way, this robust methodology eliminates the interferences that different lighting conditions or the use of different smartphones could cause. In other words, the system self-calibrates for each measurement. This study is intended as a proof of concept in which it has been demonstrated that the methodology is practical and works. The methodology has been optimized with eight prepared cooked pork shoulder samples, but additionally, a proof of concept with 18 commercial meat products has been carried out. This new procedure based on POLYSEN + APP has great potential in the quality control of meat, reducing analysis times and costs.

## Supporting Information Available

Brief data (excel file) (XLSX)
Reference method used for the determination of the residual concentration of nitrite in meat samples (based on standard ISO 2918:1975); recipe for the manufacturing of cooked pork shoulder; synthesis and characterization of *N*-(3-hydroxyphenyl)methacrylamide (HPMA); characterization of the film-shaped polymeric sensor (POLYSEN); user manual for the smartphone app “Colorimetric Titration”; statistical comparison between the reference and POLYSEN + APP methods; and commercial samples (PDF)
