# Supplementary material for: Easy Nitrite Analysis of Processed Meat with Colorimetric Polymer Sensors and a Smartphone App
Source: ACS Appl Mater Interfaces. 2022 Aug 3;14(32):37051–8. doi: 10.1021/acsami.2c09467 (PMC9389542; doi:10.1021/acsami.2c09467)
Supplement: Supplementary file 2 — am2c09467_si_002.pdf [file am2c09467_si_002.pdf]

Supporting Information (SI)

EASY NITRITE ANALYSIS OF PROCESSED MEAT WITH COLORIMETRIC POLYMER SENSORS AND A SMARTPHONE APP

Marta GUEMBE-GARCÍA[a], Lara GONZÁLEZ-CEBALLOS[a], Ana ARNAIZ[a,d], Miguel A. FERNÁNDEZ-MUIÑO[b], M. Teresa SANCHO[b], Sandra M. OSÉS[b], Saturnino IBEAS[a], Jordi ROVIRA[b], Beatriz MELERO[b], Cesar REPRESA[c], José M. GARCÍA[a,*], Saúl VALLEJOS[a,*]

[a] Departamento de Química, Facultad de Ciencias, Universidad de Burgos, Plaza de Misael Bañuelos s/n, 09001 Burgos, Spain
[b] Departamento de Biotecnología y Ciencia de los Alimentos, Universidad de Burgos, Plaza de Misael Bañuelos s/n, 09001 Burgos, Spain
[c] Departamento de Ingeniería Electromecánica, Escuela Politécnica Superior, Universidad de Burgos, Avda. Cantabria, s/n, 09006 Burgos, Spain
[b] Universidad Politécnica de Madrid, Madrid, Spain
[*] Corresponding authors: E-mail addresses: svallejos@ubu.es (S. Vallejos), jmiguel@ubu.es (J. M. García).

Table of contents

S1. Reference method used for the determination of the residual concentration of nitrite in meat samples (based on standard ISO 2918:1975)………………………………………………………… S3
S2. Recipe for the manufacturing of cooked pork shoulder…………………………………………….. S5
S3. Synthesis and characterization of *N*-(3-hydroxyphenyl)methacrylamide (HPMA)……………….. S6
S4. Characterization of the film-shaped polymeric sensor (POLYSEN)……………………………….. S7
S5. User manual for the smartphone APP "Colorimetric Titration"…………………………………… S8
S6. Limits of detection (LOD) and quantification (LOQ) S9
S7. Commercial samples S10
S8. Statistical analysis. Comparison between the reference and POLYSEN + APP methods S17

S1. Reference method used for the determination of the residual concentration of nitrite in meat samples (based on standard ISO 2918:1975)

Principle

Nitrites are extracted from the food sample, and the absorbance of the formed colour in the presence of sulfanilic acid and α-naphthylamine is read by colorimetry or spectrophotometry.

Experimental procedure

1. Extraction

Weigh about 10 g (m) of the food sample with a precision of 1 mg, and place it in a 250 mL Erlenmeyer. Add 150 mL of ethanol 40% and stir the mixture at reflux for at least one hour.

Transfer to a 250 mL volumetric flask with ethanol (40%) through a funnel, add 5 mL of each Carrez reagent, and make up to the mark. Shake and let stand for 10 min.

Centrifuge 5 min at 2000 r.p.m. and separate the excess fat with the help of a spatula. Filter into a 200 mL volumetric flask up to the mark. Pour the contents of the flask into a 500 mL beaker, scraping with a small portion of water. Place on a plate and evaporate the liquid to approximately 200 mL to remove ethanol. Allow to cool to room temperature and make up to the mark with water in a 250 mL volumetric flask.

2. Measurement

Take 25 mL of the prepared solution and add approximately 1 g of activated carbon if it is necessary to decolorize. Filter until the filtrate is clear. Take a 10 mL aliquot of the filtrate (V) and put it in a test tube. Add 10 mL of the colorimetric reagent (sulfanilic acid and α-naphtylamine), mix and allow the solution to stand in the dark for 15 min at room temperature.

After 20 min and before 4 hours, measure the optical density of the solution in a cuvette with a 1 cm light path at 520 nm wavelength. If the coloured solution is out of the curve pattern, take an aliquot of less than 10 mL. Each measurement is carried out by duplicate.

3. Curve pattern

Take aliquots of 5, 10,20, 40 and 50 mL from the standard solution containing 5 mg/L of nitrites and make up to 100 mL with water. The content of sodium nitrite in these solutions is 0.25, 0.50, 1, 2 and 2.5 mg/kg, respectively.

Transfer 10 mL of each of these solutions to a test tube and add 10 mL of the colorimetric reagent and read the absorbance at 520 nm (C).

4. Calculations

The nitrite content of the sample expressed in mg/kg can be calculated using the formula: mg/kg $NaNO_2$ = C*(2500/mV), where m = food sample weight; V = volume, in mL taken from the decoloured extract; and C = concentration of sodium nitrite expressed in mg/kg determined on the standard curve.

5. Remarks

The difference between two parallel determinations of the same sample, carried out simultaneously by the same analyst, must not exceed 10% of the calculated nitrite content.

6. Standard

ISO/DIS 2918

S2. Recipe for the manufacturing of cooked pork shoulder

Table S1. List of ingredients for the preparation of the starting mix.

Ingredients	Weight %	Grams
Pork shoulder	72.92	5,833.40
Sodium chloride	1.74	138.84
Sodium Tripolyphosphate	0.51	40.43
Sodium Caseinate	1.12	89.83
Smoked meat flavour	0.22	17.98
Water	23.49	1,879.52
	**TOTAL**	**8,000.00**

This mass was split into 8 batches of 1 kg, and finally, different amounts of sodium nitrite, sodium chloride, carrageenan, starch and water were added to each batch, mixed and sealed under vacuum.

Table S2. List of ingredients for the preparation of 5 calibration samples (C1-C5) and 3 test samples (T1-T3).

	**Calibration Samples**					**Test Samples**		
**Sample Name**	**C1**	**C2**	**C3**	**C4**	**C5**	**T1**	**T2**	**T3**
Meat-Starting Mix (g)	1000.0	1000.0	1000.0	1000.0	1000.0	1000.0	1000.0	1000.0
Sodium chloride (g)	5.0	5.0	5.0	5.0	5.0	5.0	5.0	5.0
Carrageenan (g)	5.5	5.5	5.5	5.5	5.5	5.5	5.5	5.5
Starch (g)	5.5	5.5	5.5	5.5	5.5	5.5	5.5	5.5
Water (g)	100.0	100.0	100.0	100.0	100.0	100.0	100.0	100.0
Sodium nitrite (mg)	1.5	57.8	173.3	231.1	462.2	462.2	57.8	1.5
**Manufacturing [NO2]-**	**1.0**	**37.5**	**112.5**	**150.0**	**300.0**	**300.0**	**37.5**	**1.0**

S3. Synthesis and characterization of *N*-(3-hydroxyphenyl)methacrylamide (HPMA)

Synthesis of N-(3-hydroxyphenyl)methacrylamide (HPMA). 2.68 g (24.56 mmol) of 3-aminophenol and 3.85 mL (3.98 g, 25.79 mmol) of methacrylic anhydride were added to a pressure flask, and the mixture was stirred at 100ºC for 2 hours. The mixture was cooled to room temperature, and hexane (50 mL) was added to the flask. The pure product HPMA (4.23 g) was filtered and dried at air. Yield: 97%. ^{1}H NMR (300 MHz, DMSO-*d*$_6$) δ (ppm) = 9.64 (s, 1H), 9.36 (s, 1H), 7.28 (s, 1H), 7.08 (d, J=5.13, 2H), 6.49 (m, 1H), 5.78 (s, 1H), 5.48 (s, 1H), 1.95 (s, 1H). ^{13}C NMR (75.5 MHz, DMSO-*d*$_6$) δ (ppm) = 167.23, 157.91, 141.00, 140.54, 129.58, 120.15, 111.42, 111.05, 107.78, 19.23. HRMS (EI) m/z [M+H]+ calc.:178.0790; found:178.0866.

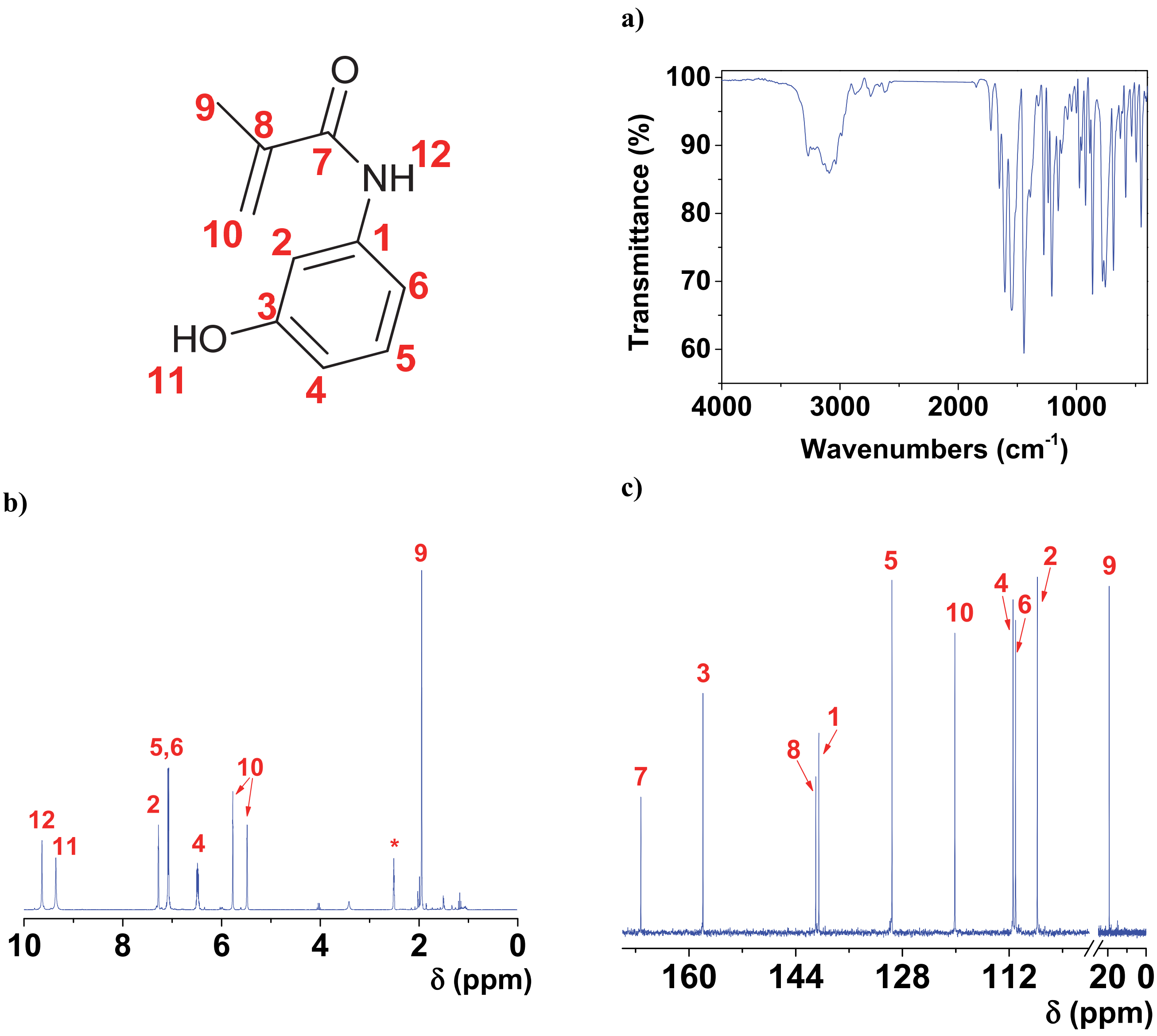

Figure S1. Characterization of *(1)* by (a) FTIR, (b) 1H RMN, and (c) 13C RMN. (* = solvent signal, CDCl3).

S4. Characterization of the film-shaped polymeric sensor (POLYSEN)

a)

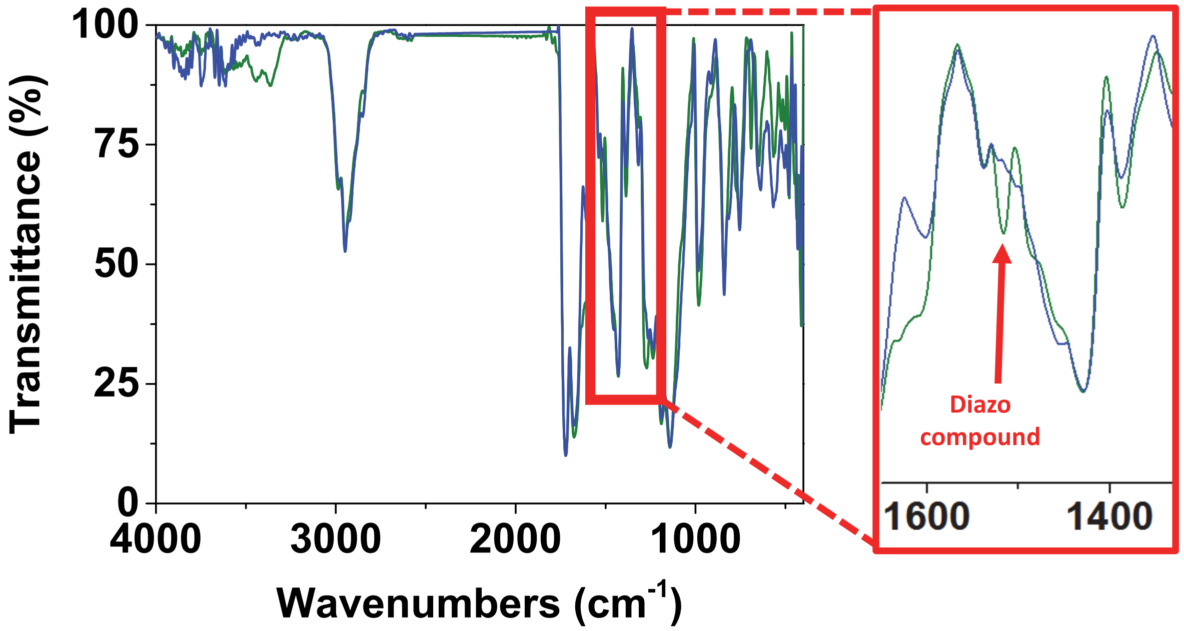

b)

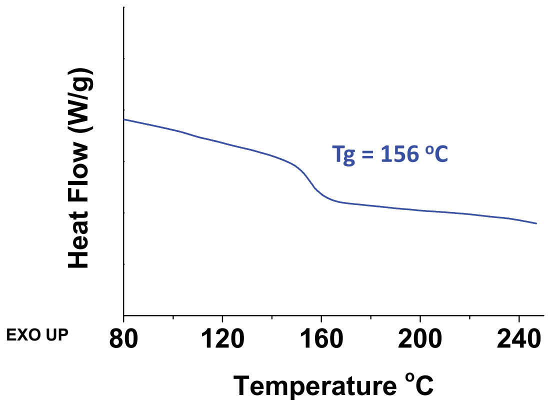

c)

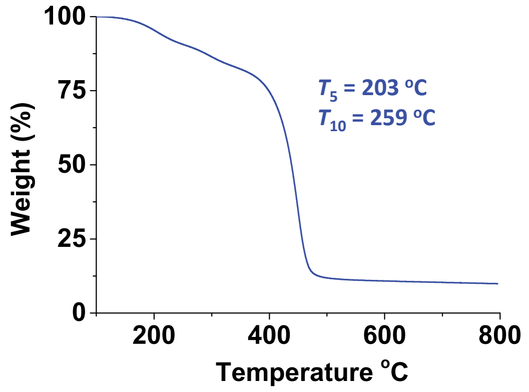

d)

Young´s Modulus =573 MPa

e)

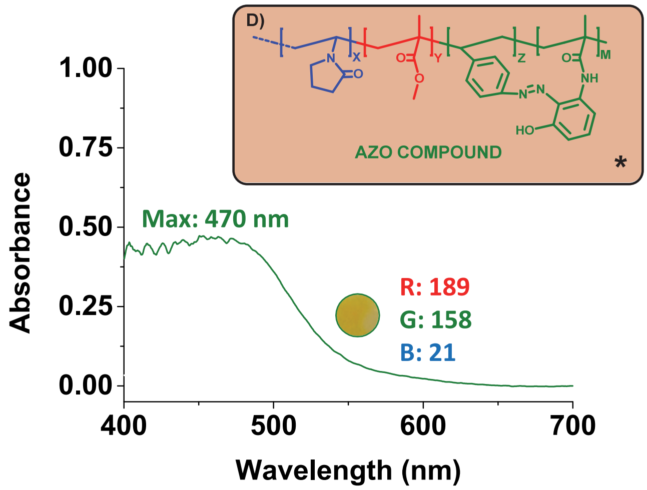

Figure S2. Characterization of POLYSEN by (**a**) FTIR, Figure shows the IR spectra of POLYSEN before (blue) and after (green) detection of nitrite. Zoom window shows the band of the formed diazo compound at 1516 cm^{-1} (**b**) DSC curve at a heating rate of 20 °C·min^{-1} under nitrogen atmosphere. The figure shows the glass transition temperature (*T*g) at 156 ºC. (**c**) Thermogravimetric curves at 10 °C·min^{-1} under nitrogen atmosphere. The figure shows T_5 and T_{10} values, the temperatures at which 5% and 10% of the weight is lost, respectively. (**d**) Tensile properties of the material, Young's Modulus =573 MPa. (**e**) UV-Vis spectrometry spectra of a calibration sample (112.5 mg/kg of manufacturing [NO_2^-]) after azo-coupling reaction, including polymer's formula and real photograph of the film. An optic fibre accessory was used for recording the absorbance spectra.

S5. User manual for the smartphone APP "Colorimetric Titration".

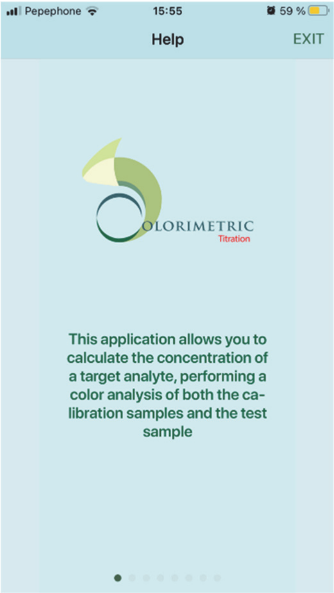

a) Home page of the "help" tab

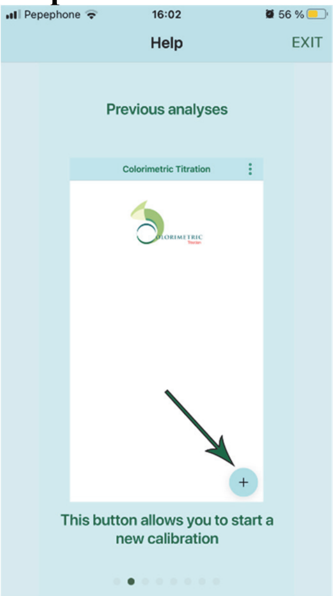

b) The first step is to create a new analysis, although the application saves the history of previous analysis and allows the editing of points and graphs previously performed.
The photo can be uploaded from the smartphone library or can be taken through the APP.

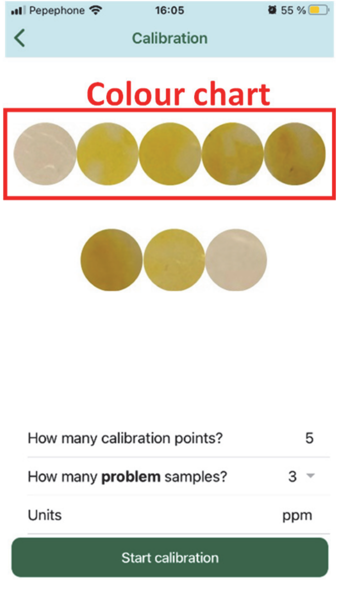

c) In the second step, the user must enter the number of calibration samples (calibration colour chart) and the number of test samples, as well as the concentration units.

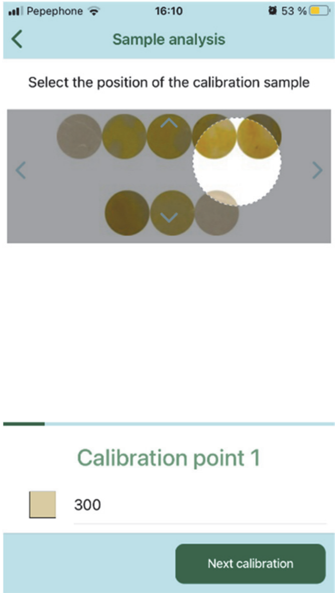

d) Next, the user must adapt the circular selector in each of the test and calibration samples since the application will read the colour parameters within the circle. Additionally, and only for calibration samples, the user must indicate the concentration of each sample.

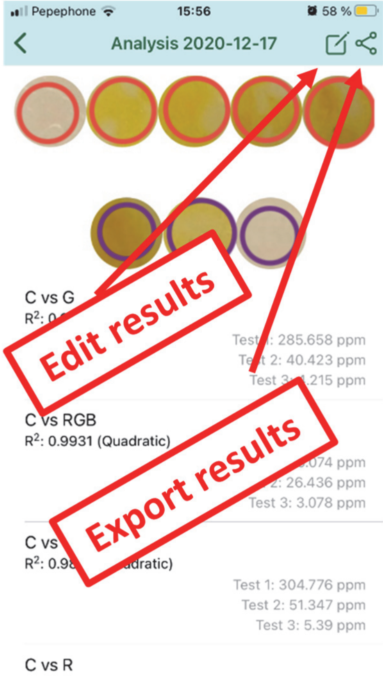

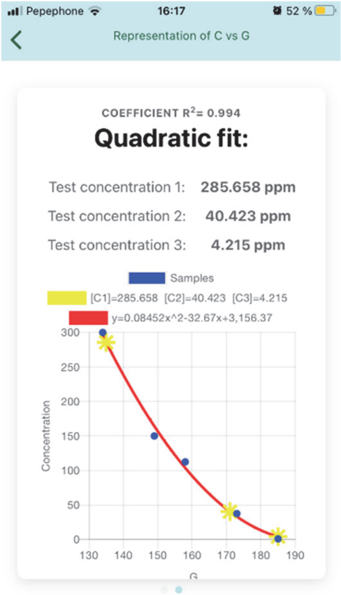

e) In the next window, the application shows the result of the analysis, which can be exported by email in CSV format. The application will perform the graphical representation of the concentration (and logarithm of concentration) against different colour parameters (red R, green G, blue B, hue H, saturation S, value V, principal components of RGB and ΔRGB. Additionally, the APP performs linear and quadratic fits, a total of 32 fits. The fitted curve with the highest R^2 parameter will be considered as the best option and will appear in the first position

f) Graph view of analysis. Calibration points are shown in blue, and test samples in yellow.

S5. Limits of detection (LOD) and quantification (LOQ)

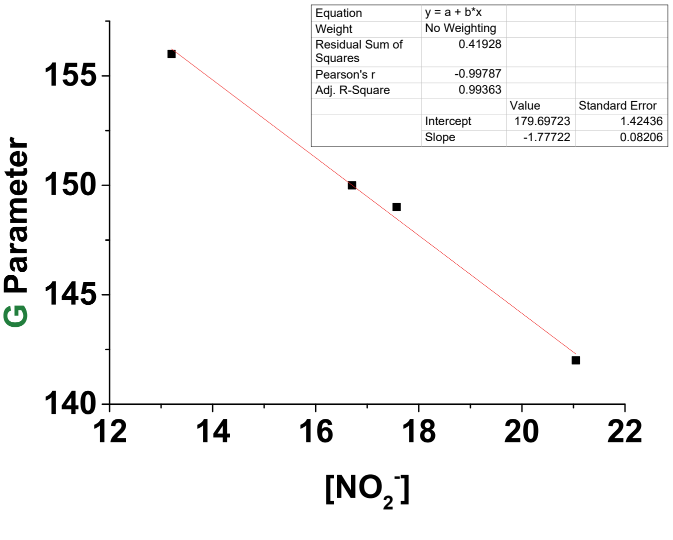

Figure S3. Graphical representation of the G parameter of the photographed discs vs [NO_2^-] at low concentrations, showing the equation for the fitted line, which allows the calculation of the LOD and the LOQ.

Table S3. Data used for the shown graph in Figure SX.

[NO_2^-]	G parameter
13.2	156
16.7	150
17.6	149
21.0	142

S7. Commercial samples

Table S4. Commercial samples used in case study A. Table shows the product's image, the manufacturer, the list of ingredients, the nitrite concentration obtained by the reference method, the image of the POLYSEN calibration and test discs, and the obtained G parameter. The rest of the digital colour parameters can be found in the excel file **ESI-APP-DATA**. Nitrite concentration data from reference and POLYSEN+APP methods are means of ± standard deviation of 2 and 3 replicates, respectively.

CASE STUDY A	Manufacturer (Commercial name)	Composition/Ingredients	Nitrite concentration by reference method (mg/kg)	POLYSEN sensory disc after the analysis	Obtained G parameter from the photograph	Nitrite concentration by POLYSEN + APP method (mg/kg)
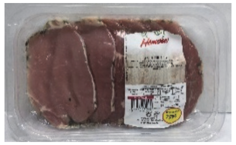	Hermosa (Garlic tenderloin)	Pork loin, salt, sugar, antioxidant (E-301, E-331iii), preservative (), pork protein, spices. gluten-free.	0.57 ± 0.48	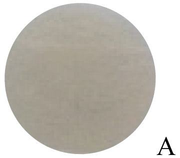 A	163.33 ± 0.58	1.87 ± 0.98
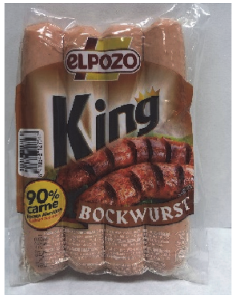	El Pozo (King Bockwurst)	Pork (90%), water, salt, soy protein, sugar, paprika, spices, aromas and smoke aroma, stabilizer: (E-451), antioxidant: (E-316, E-331), preservative: (E-250) and dye: (E-120). It may contain traces of milk.	9.94 ± 0.06	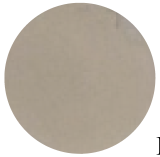 B	159.33 ± 1.15	7.96 ± 1.56
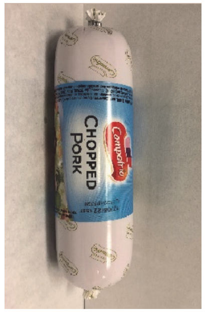	Campofrío (Chopped pork)	Pork (53%), water, mechanically separated chicken meat, pork fat, starch, salt, dextrose, stabilizers (E451, E450, E452, E407a, E412, E508), flavourings, antioxidant (E316), preservative (E250).	13.20 ± 0.36	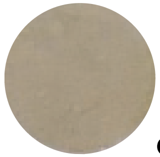 C	156.00 ± 1.00	12.13 ± 1.12

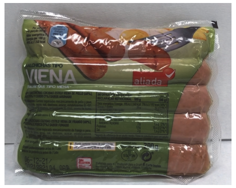	Aliada (Vienna sausage)	Mechanically separated chicken and turkey meats, pork fat, water, salt, dextrose, sugar, stabilizers (E-451, E-466), spices and aromas, antioxidant (E-316), smoke aroma, preservative (E- 250) May contain traces of soy and milk.	13.74 ± 0.00	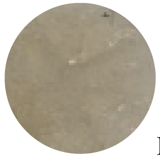 D	153.67	14.44 ± 2.07
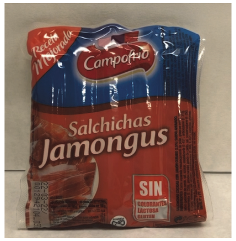	Campofrío (Jamongus sausages)	Mechanically separated meat from chicken, pork fat, water, bacon [pork belly, salt, sugar, stabilizer (E-451), antioxidant (E-316), preservative (E250)], cured ham (3.5%) [pork ham, salt, preservatives (E-250, E-252)], dextrose, salt, starch, stabilizers (E-466, E-451), spices, aromas, antioxidant (E-316), smoke aroma, conservative (E-250). Coating: collagen.	16.70 ± 0.29	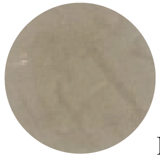 E	150.00 ± 2	17.44 ± 1.34
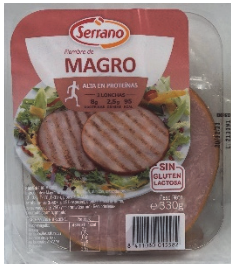	Serrano (Lean pork)	Lean pork (47%), water, starch, antioxidants (E-326, E-331, E-316), soy protein, salt, dextrose, sugar, stabilizers (E-407, E451), spices, aromas, smoke aroma, preservatives (E-250) and dye (carmine).	17.57 ± 018	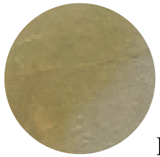 F	149.00 ± 6.56	17.08 ± 3.64
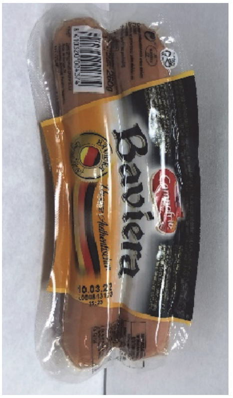	Campofrío (Baviera sausage)	Pork meat and fat, mechanically separated meat from chicken, water, pork rinds, starch, salt, sugar, spices and flavourings, stabilizers (E-452, E-450 and E-338), antioxidant (E-316) and conservative (E-250). It may contain traces of soy and milk.	21.05 ± 0.06	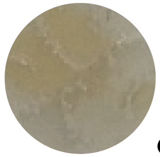 G	142.00 ± 5.57	19.69 ± 0.61

**TEST SAMPLE** 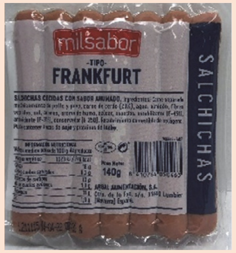	Milsabor (Frankfurt sausage)	Mechanically separated meat from chicken and turkey, pork (23%), water, starch, vegetable fibres, salt, flavourings, smoke flavourings, sugar, stabilizing species (E-451), antioxidants (E-316), preservative (E-250). Edible collagen coating. It may contain traces of soy and milk proteins.	17.02 ± 0.36	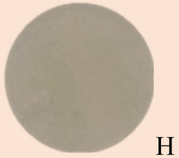 H	149.67 ± 1.53	17.70 ± 0.96

Table S5. Commercial samples used in case study B. Table shows the product's image, the manufacturer, the list of ingredients, the nitrite concentration obtained by the reference method, the image of the POLYSEN calibration and test discs, and the obtained G parameter. The rest of the digital colour parameters can be found in the excel file **ESI-APP-DATA**. Nitrite concentration data from reference and POLYSEN+APP methods are means of ± standard deviation of 2 and 3 replicates, respectively.

CASE STUDY B	Manufacturer (Commercial name)	Composition/Ingredients	Nitrite concentration by reference method (mg/kg)	POLYSEN sensory disc after the analysis	Obtained H parameter from the photograph	Nitrite concentration by POLYSEN + APP method (mg/kg)
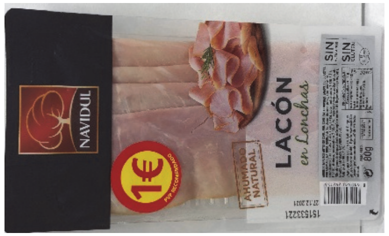	Navidul (Sliced pork shoulder)	Pork ham (85%), water, antioxidants (E-325, E-316, E-331iii), salt, dextrose, stabilizers (E-451i, E-452, E-407), sugar, preservatives (E- 262, E-243, E-250), spices, aromas, and hydrolyzed vegetable protein.	3.16 ± 0.12	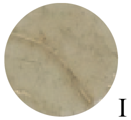 I	0.106 ± 0.001	4.70 ± 0.85
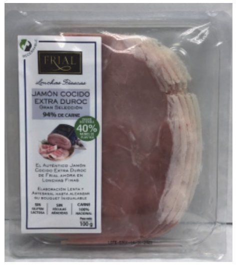	Frial (extra Duroc cooked ham)	Duroc boneless pork ham (94%), water, antioxidants (E-326, E-301), salt, flavourings, dextrose, spices, stabilizers (E-451), preservatives (E-261, E-250). It may contain soy.	3.36 ± 0.06	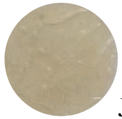 J	0.105 ± 0.001	4.21 ± 0.85
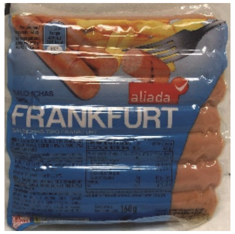	Aliada (Frankfurt sausages)	Mechanically separated meat from chicken, fat and pork rind, mechanically separated meat from turkey, water, starch, salt, sugar, stabilizers (E-451, E-415, E-412), spices, antioxidant (E-316), smoke aroma, preservative (E-250). It May contain traces of soy and milk	6.94 ± 0.17	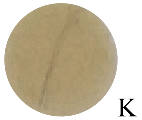 K	0.106 ± 0.001	5.67 ± 0.85

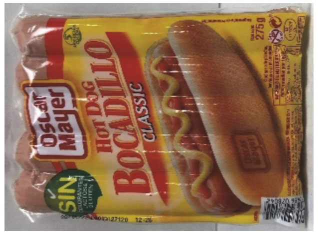	Oscar Mayer (Hot Dog sandwich classic)	Mechanically separated meat from chicken, pork fat, water, salt, dextrose, stabilizers (E-466, E-508, E-450), aromas and spices, smoke aroma, antioxidant (E-316) preservative (E-250). It may contain traces of soy and milk protein.	15.40 ± 0.06	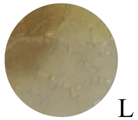 L	0.112 ± 0.002	13.49 ± 3.05
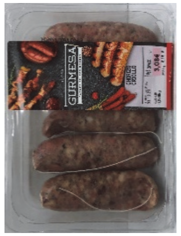	Gurmesa (Creole sausage)	Pork, pork fat, water, salt, spices, dextrose, sucrose, pea protein, dextrin, emulsifiers (E-450, E-451), antioxidant (E-310), preservatives (E-250, E- 252, E262ii), pork protein, gluten-free fibres, flavourings, vegetable extracts, acidulants (E-296), dye (E-120) and natural casing. It can contain traces of soy and milk derivatives.	15.44 ± 0.23	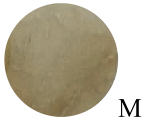 M	0.114 ± 0.003	17.46 ± 4.39
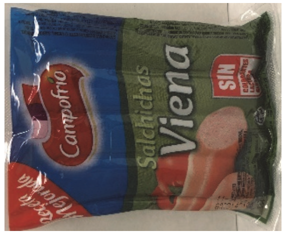	Campofrío (Vienna sausage)	Mechanically separated meat from chicken, mechanically separated meat from turkey, pork fat, dextrose, starch, salt, water, spices, stabilizer (E-451), antioxidant (E-316), smoke aroma, preservative (E-250). Coating: collagen.	17.11 ± 0.17	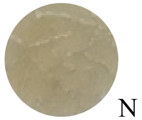 N	0.113 ± 0.002	15.45 ± 2.54
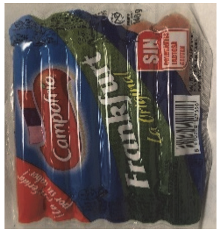	Campofrío (Frankfurt sausages)	Mechanically separated meat from chicken, pork fat, water, dextrose, salt, starch, stabilizers (E-466, E-451), spices and aromas, antioxidant (E-316), smoke aroma, preservative (E-250). Coating: collagen. It may contain traces of soy and milk proteins.	17.74 ± 0.06	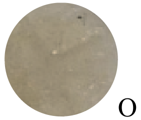 O	0.114 ± 0.002	16.92 ± 2.93

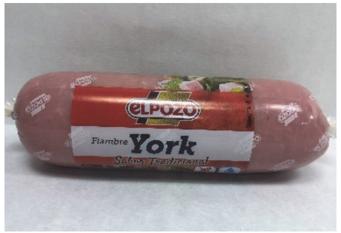	El Pozo (Stiff york)	Stabilizers: E-420, E-450 and E-407. Conservator: E-250. Antioxidant: E-316. Dye E-120. Contains traces of milk protein.	19.99 ± 0.24	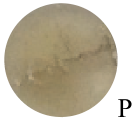 P	0.117 ± 0.001	21.31 ± 1.47
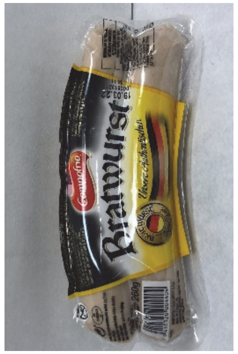	Campofrío (Bratwurst sausages)	Cooked pork sausages (86%): Pork meat and fat, water, starch, salt, sugar, stabilizers (E-451, E-450), flavourings and spices.	29.68 ± 0.23	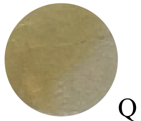 Q	0.123 ± 0.003	29.62 ± 3.69

**TEST SAMPLE** 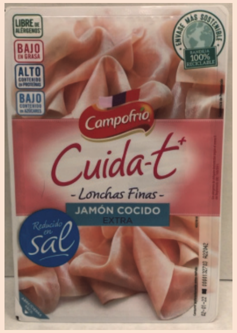	Campofrío (Cooked ham Cuida-t+)	Pork ham (75%), water, flavourings, stabilizers (E-450, E-508, E-407a, E-412), salt, dextrose, sugar, antioxidant (E-316) and preservatives (E-262, E-250).	5.32 ± 0.06	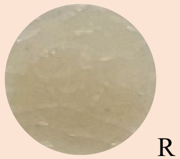 R	0.107 ± 0.001	6.65 ± 1.47

S8. Statistical analysis. Comparison between Reference and POLYSEN + APP methods.

This procedure is designed to compare two data samples and will determine if there are statistically significant differences between the two methods.

A) Description of the variables

First of all, the normal distribution of data must be checked. **Table S6** contains the statistical summary for the two data samples. Since the values for "standardized bias" and "standardized kurtosis" are out of the range -2 to 2, normal distributions of the data cannot be assumed. Therefore, a Wilcoxon matched-pairs signed rank test was performed.

Table S6. Statistical Summary

	**Reference Method** (standard ISO 2918:1975)	**POLYSEN + APP method**
**Count**	21	21
**Mean**	17.46	17.52
**Standard deviation**	22.09	21.89
**Coefficient of variation**	126.52%	124.98%
**Minimum**	0.57	1.87
**Maximum**	108.62	108.01
**Range**	108.05	106.14
**Standardized Bias**	7.12	7.17
**Standardized Kurtosis**	15.10	15.24

B) Paired t-test and correlation test

A paired test and a correlation test for non-normal distributed data have been carried out to analyze whether the data obtained with the reference and the POLYSEN+APP methods are comparable and do not present significant differences between them. **Table S7** shows the results from two tests. Wilcoxon matched-pairs signed rank analysis indicates that there are no significant differences between the data obtained with both methods since the p-value is greater than 0.05. On the other hand, the Spearman test shows that the data obtained with both methods are statistically correlated in a significant way, with a p-value less than 0.0001 and an rs value of 0.9519.

Table S7. Wilcoxon matched-pairs signed rank test and Spearman correlation test.

**P value**	0.8117
**Exact or approximate P value?**	Exact
**P value summary**	ns
**Significantly different (P < 0.05)?**	No
**One- or two-tailed P value?**	Two-tailed
**Sum of positive, negative ranks**	123.0 , -108.0
**Sum of signed ranks (W)**	15
**Number of pairs**	21
**Number of ties (ignored)**	0
**How effective was the pairing?**	
**rs (Spearman)**	0.9519
**P value (one tailed)**	<0.0001
**P value summary**	****
**Was the pairing significantly effective?**	Yes
**Was the pairing significantly effective?**	Yes
